# Portal vein embolization versus radiation lobectomy as pre-treatment for major liver resection for colorectal liver metastases: functional assessment of the future liver remnant

**DOI:** 10.1007/s10456-026-10049-5

**Published:** 2026-05-20

**Authors:** Khalil Ramdhani, Rosalie C. M. van Rees, Daan Andel, Jeanine M. L. Roodhart, Arthur J. A. T. Braat, Rutger C. G. Bruijnen, Inne H. M. Borel Rinkes, Onno Kranenburg, Marnix G. E. H. Lam, Maarten L. J. Smits, Jeroen Hagendoorn

**Affiliations:** 1 Department of Radiology and Nuclear Medicine, Cancer Center Utrecht, University Medical Center Utrecht, Utrecht, The Netherlands; 2 Department of Surgical Oncology, Cancer Center, University Medical Center Utrecht, Heidelberglaan 100, 3584 CX Utrecht, The Netherlands; 3 Laboratory Translational Oncology, Division of Imaging and Cancer, Department of Surgical Oncology, University Medical Center Utrecht, Utrecht, The Netherlands; 4 Department of Medical Oncology, University Medical Center Utrecht, Utrecht, The Netherlands; 5 Department of Nuclear Medicine, Netherlands Cancer Institute, Amsterdam, The Netherlands

**Keywords:** Radioembolization, Portal vein embolization, 99mTc-mebrofenin hepatobiliary scintigraphy, Colorectal tumor metastases, Future liver remnant

## Abstract

**Introduction:**

Insufficiency of the future liver remnant (FLR) precludes surgery for liver tumors as it is associated with post-hepatectomy liver failure (PHLF). A common strategy to induce pre-operative FLR hypertrophy is portal vein embolization of the tumor bearing liver lobe. More recently, transarterial radio-embolization (radiation lobectomy, RL) has been employed. Direct functional assessment using 99mTc-mebrofenin-hepatobiliary-scintigraphy (HBS) predicts FLR sufficiency more accurately than conventional volume assessment. However, studies describing dynamic functional assessment of the FLR after RL as induction for surgery are currently lacking. This study aims to compare FLR functional changes after PVE and RL.

**Methods:**

This non-interventional retrospective single-center cohort study was performed between 2016 and 2024. Patients with colorectal liver metastases (CRLM) who underwent PVE or RL because of an insufficient FLR (HBS < 2.7%/min/m^2^) were included. Induction of sufficient FLR function was the primary outcome.

**Results:**

Ten PVE- and ten RL-treated patients were included. The median duration to achieve sufficient FLR function was longer for RL- than PVE-treated patients (75 days (64–85) vs 31 days (28–54.5), *p* = 0.002). RL showed a non-significant higher median functional increase (58.6% vs 51.1%, *p* = 0.940) and a significantly higher voluminal increase (65.7% vs 36.8%, *p* = 0.049) compared to PVE. PHLF and resection margins were comparable among groups. There was no 90 day mortality.

**Conclusion:**

RL represents a feasible alternative to PVE with comparable functional outcomes, particularly in patients with lower baseline FLR function or need for simultaneous tumor control. This warrants prospective studies with optimized RL protocols to better define the functional outcomes and clinical applications of RL versus PVE.

**Supplementary Information:**

The online version contains supplementary material available at 10.1007/s10456-026-10049-5.

## Introduction

Liver resection can cure primary liver cancer and metastatic liver disease. However, an insufficient future liver remnant (FLR) precludes liver resection as it is associated with an increased risk of post-hepatectomy liver failure (PHFL) and mortality rates of up to 21% [1–5]. CT-based volumetry has been the gold standard for the assessment of FLR sufficiency and the identification of those who are at risk of developing PHLF [2, 3, 6]. The minimum volume required to obtain adequate post-surgical liver function depends on several factors such as underlying quality of the liver and tumor contribution, making this technique prone to errors [7, 8]. Over the past years, studies demonstrated the superiority of functional over voluminal assessment using ^99m^Tc-mebrofenin-hepatobiliary-scintigraphy (HBS) to predict PHLF, maintaining a cut-off value of 2.7%/min/m^2^ [9, 10].

**Fig. 1:**
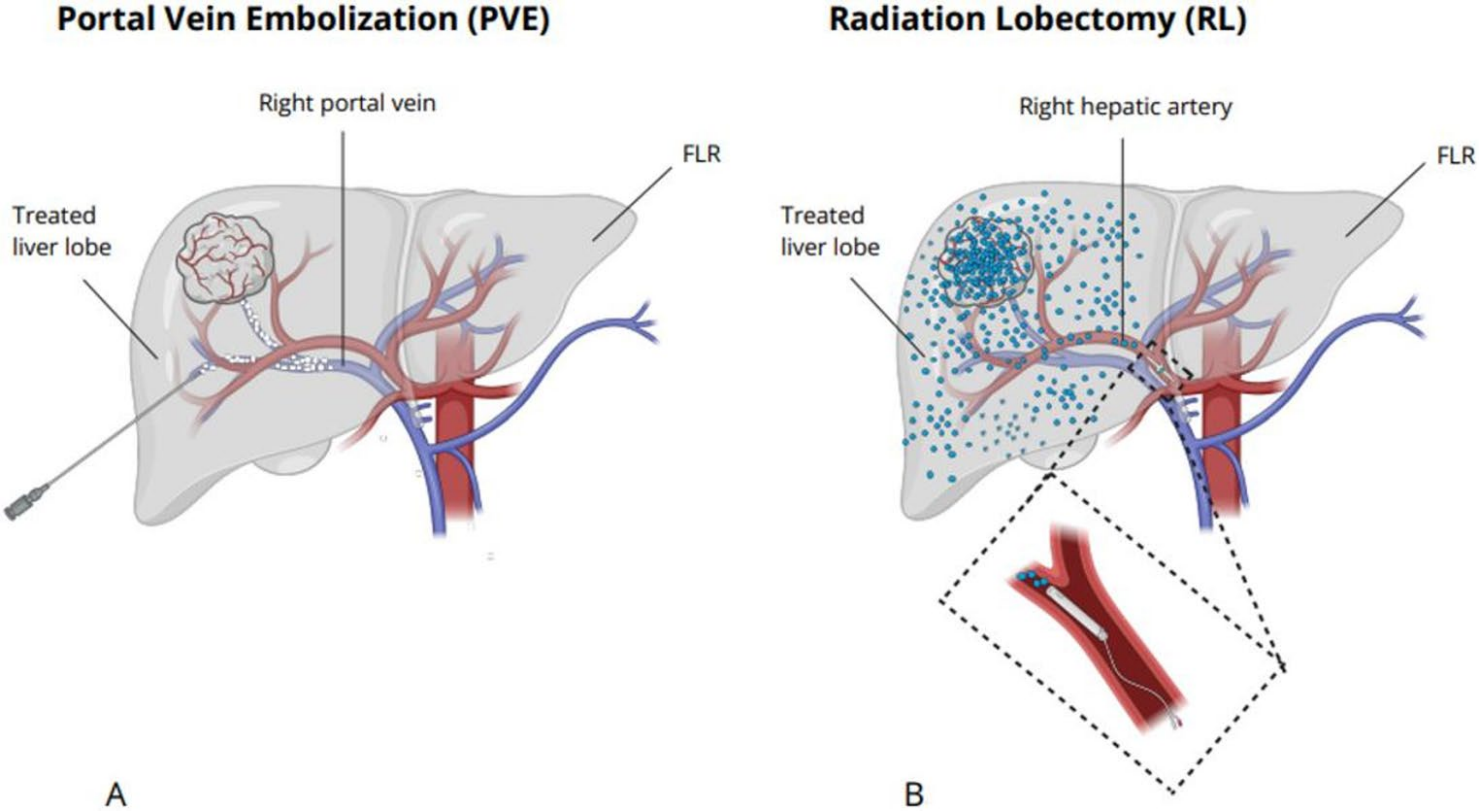
Schematic illustration of **A** Portal vein embolization (PVE) and **B** Radiation lobectomy (RL)

The liver has a distinctive dual blood supply, via the portal vein and the hepatic artery. Portal vein embolization (PVE) is widely used to induce hypertrophy when FLR is insufficient [8, 11]. Embolization of branches of the portal vein (of the tumor bearing liver lobe) causes shunting of the portal blood supply to the contralateral lobe, thereby inducing hypertrophy (Figure 1A). FLR volume increases with a mean of 37.9% (± 0.1%) within a mean response interval of 25.9 days (± 10.1) [12]. However, FLR growth takes place almost exclusively in the first three weeks after treatment and reaches a plateau phase thereafter [13]. In addition, tumor progression can occur during this period, and its effect on tumor growth is unclear [11, 14]. Moreover, Cieslak et al. [15] demonstrated that patients without neoadjuvant chemotherapy require a baseline FLR function of ≥ 1.72%/min/ m^2^ to achieve adequate FLR function post-PVE.

Radiation lobectomy (RL) is an alternative procedure to increase the FLR. RL uses high-dose transarterial radioactive microspheres to irradiate the tumor and adjacent liver tissue, causing contralateral hypertrophy (with reported hypertrophy rates of 26–45% over 44 days to 9 months) (Figure 1B) [16–19]. RL-induced growth is slower but more prolonged than PVE, with functional changes observed up to nine months [13, 17]. Moreover, RL offers additional benefits; ipsilateral irradiation allows local tumor control during the response period and causes tumor necrosis, which may facilitate tumor-free resection [8, 20]. Moreover, the waiting period may serve as a “test-of-time” since it enables the identification of new lesions which may contra-indicate surgery [20].

Previous studies comparing induction of the FLR after both treatment strategies are rare and outcomes were not consistent [21, 22]. These studies focused on voluminal changes whereas FLR functional increase more precisely predicts PHLF [10]. In addition, FLR function increases more significantly compared to volume, which may prevent unnecessary waiting time prior to surgery [23]. Therefore, this retrospective cohort study aims to assess the functional increase of the FLR in response to PVE and RL.

## Methods

### Study design and patients

A non-interventional retrospective cohort study was performed in the University Medical Center Utrecht (UMCU) from 2016 to 2024. Institutional board approval was obtained and the need for written informed consent was waived. Patients were included if they were diagnosed with colorectal liver metastases (CRLM), had an insufficient FLR and required treatment with either PVE or RL with the intent to increase the FLR in preparation for surgery. Decisions regarding treatment strategy to induce FLR hypertrophy were all made in a multidisciplinary tumor board. In general, PVE was considered the standard approach for induction of the FLR. RL was selectively applied in specific clinical scenarios: when PVE was technically not feasible, as “test of time” strategy to assess tumor biology and in patients who were not eligible for systemic therapy. HBS had to be performed before and after FLR induction therapy. Insufficiency of the FLR was defined as function < 2.7%/min/m^2^ measured with HBS, there was no minimal FLR function warranted.

### Imaging

^99m^Tc-mebrofenin was used to perform HBS similar as previously described [24]. After administration of 200 MBq ^99m^ Tc-mebrofenin, a dynamic acquisition (both in anterior and posterior direction) was made using the Symbia T16 SPECT/CT (Siemens) to evaluate hepatic uptake, which was corrected for body surface area (%/min/m^2^). This was followed by performance of a fast SPECT/CT, enabling 3D-assessment of the total liver function. At the same time, a low dose CT-scan was made for attenuation correcting and a diagnostic contrast enhanced CT (CECT) was made for anatomic reference. Regions of interests were manually delineated on fused SPECT/CECT using Simplicity^®^, to calculate segmental function. In addition, these scans and regions of interests enabled volume assessment as well. HBS was performed before and after treatment with PVE or RL. Several outcome measures were used to evaluate the effectiveness of both treatment options: absolute increase, degree of functional increase, degree of voluminal increase, functional growth rate per day and voluminal growth rate per day. The degree of increase was calculated using the formula *((post-treatment FLR– pre-treatment FLR) / pre-treatment FLR) x 100%)* for FLR function and volume. For growth rate per day, the absolute increase was divided by the number of days in between treatment and sufficient FLR.

### Portal vein embolization

Portal vein embolization was performed under general anesthesia or sedation.

**Fig. 2:**
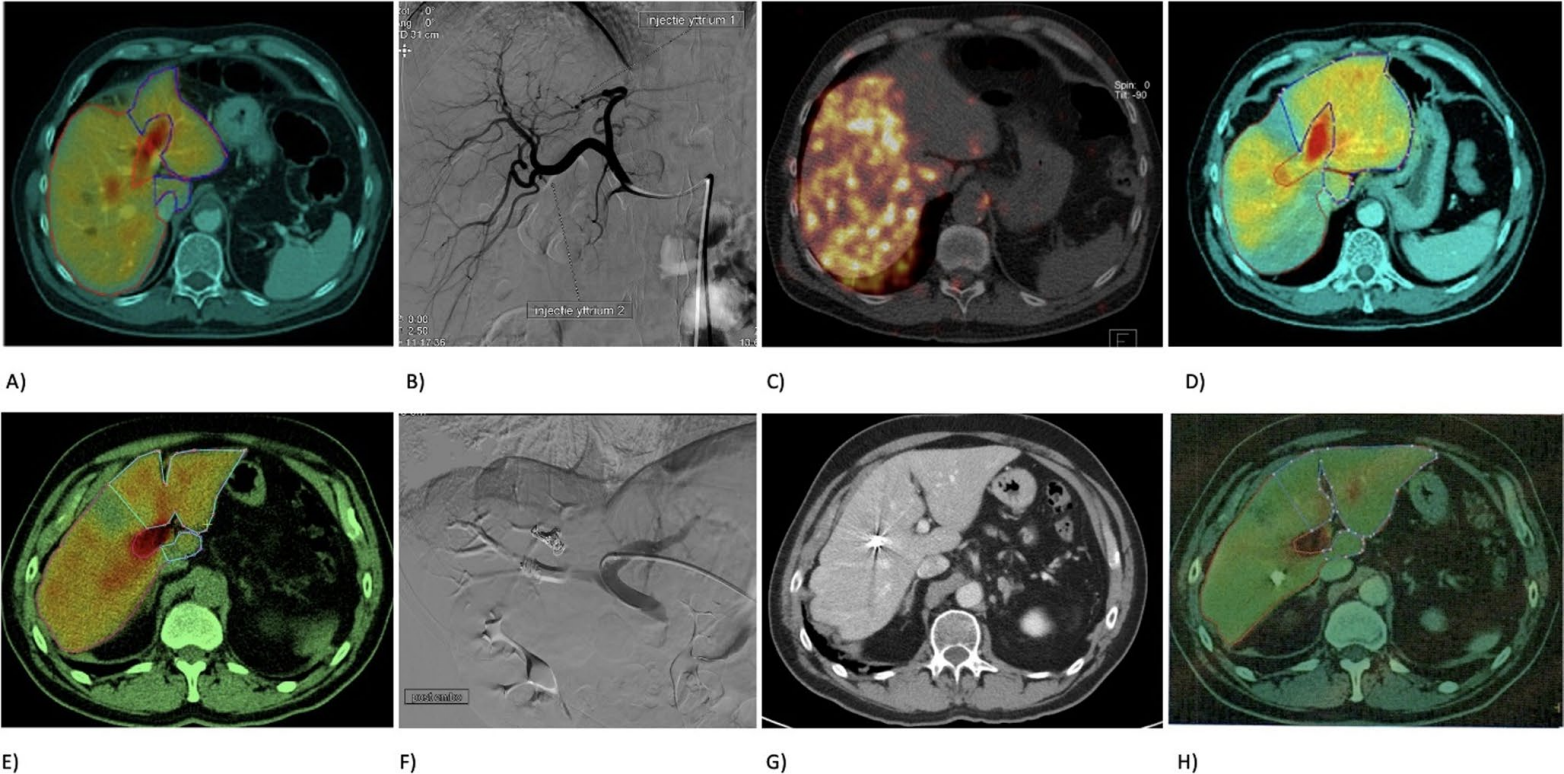
Pictorial overview demonstrating RL (**A**–**D**) and PVE (**E**–**H**). RL: **A** patient with low baseline FLR function (1.2%/min/m^2^) on HBS. **B** Scout angiography with intra-arterial ^99m^Tc-MAA injection; SPECT-CT shows accurate right-lobe targeting without extrahepatic targeting. **C** RL performed according to MAA injection; Y90 PET-CT confirms accurate delivery. **D** Two months later, HBS shows adequate FLR hypertrophy and function (3.0%/min/m^2^). PVE: **E** patient with baseline FLR function of 2.4%/min/m^2^. **F** PVE of segments 5–8 using particles, coils, and Amplatzer plug. **G** CT after one month demonstrates hypertrophy of segments 1–4. **H** HBS confirms sufficient FLR function (3.6%/min/m^2^)

The portal vein was percutaneously punctured via a transhepatic ipsi- or contralateral approach using ultrasound and fluoroscopic guidance. Embolization of the portal branches of the target segments was performed with either *N*-butyl cyanoacrylate glue (Glubran2, GEM, Srl, Viareggio, Italy) mixed with ethiodized oil (Lipiodol; Guerbet, France) or poly-vinyl alcohol particles (Boston Scientific, USA) (Figure 2). Additional coils, vascular plugs or hepatic vein embolization were used depending on the operator’s preference.

### Radiation lobectomy

To evaluate intrahepatic activity deposition in the liver, possible extrahepatic deposition and to calculate the amount of liver–lung shunting, a workup procedure was performed. During this workup procedure an angiography was performed to map the vascular system and to evaluate the catheter placement for administration of the microspheres. Subsequently a diagnostic radiotracer (a low dose of ^99m^Tc-MAA or ^166^Ho-microspheres) was intra-arterially injected to evaluate intrahepatic distribution of the microspheres and to rule out extrahepatic deposition of activity. Patients who were treated with ^166^Ho received ^166^Ho-scout microspheres (QuiremSpheres^®^, Quirem Medical/Terumo) and ^99m^Tc microalbumin aggregates were used for those who received ^90^Y-glass microspheres (Therasphere ^®^, Boston Scientific). The activity required for radiation lobectomy was adjusted to the volume of the liver targeted for treatment.

*Resection*: All patients were discussed in a multidisciplinary tumor board. An FLR function of at least 2.7%/min/m^2^ measured on HBS was deemed sufficient for major hepatic resection. Patients were considered ineligible for surgery in case of new malignant extrahepatic lesions or new lesions located in the FLR. In case of borderline FLR function, a patient-specific decision was made by experienced hepatobiliary surgeons.

### Follow-up

CT or MRI of the liver in combination with HBS were performed every month (PVE) or 2–3 months (RL) after treatment to evaluate resectability. After surgery, PHLF was graded in accordance with the International Study Group of Liver Surgery [25]. This definition of PHLF is based on elevated levels of both bilirubin and INR on postoperative day 5 or later. Increased prothrombin time (PT) was used when INR was not available. Resection margins were examined by a pathologist, distinguishing between R0 (> 1 mm tumor free margin, microscopically), R1 (< 1 mm tumor free margin, microscopically) or R2 (no tumor free margin, macroscopically).

### Statistics

Statistical analyses were performed using IBM SPSS Statistics 29^®^. Median values in combination with interquartile range (IQR) were used to annotate continuous variables. As there are low numbers of patients in this cohort, a Mann-Whitney U test was applied to assess the differences between groups (PVE versus RL). For the evaluation of differences over time in the same group, a paired t-test was performed. Chi-square tests assessed binary outcome variables; the Fisher’s exact test was used in case of expected cell frequencies lower than 5 in more than 25% cells. For correlations between continues variables, Pearson’s correlation coefficient was used.

## Results

### Patient demographics

Ten patients with CRLM treated with RL and eleven patients with CLRM treated with PVE, prior to surgery, met inclusion criteria. One of the PVE-treated patients was diagnosed with underlying liver disease (hemochromatosis). This patient was excluded to preserve homogeneity among groups as there were no diagnoses causing affected liver function in the RL-group. Eventually, this resulted in a cohort of 20 patients, each group consisting of 10 patients (supplementary Tables 1 and 2). One patient in the PVE cohort was treated with hepatic vein embolization (HVE) prior to PVE. There were no significant differences in baseline criteria among groups (Table 1). Median age at treatment was comparable among groups; 65.5 years (IQR 58.8–72.5) in the PVE-group and 59.5 years (IQR 50.3–68.8) in the RL-group (*p* = 0.256). In both groups, the median Charlson Comorbidity Index (CCI) was 8 (*p* = 0.504) and in both groups, 90% of the patients received pre-operative chemotherapy. The median largest liver lesion differed non-significantly between groups and was smaller in PVE treated patients (23.0 mm (IQR 9.6–47.4) in PVE-treated patients and 45.6 mm (IQR 30.8–67.7) in RL-treated patients (*p* = 0.059)). The median pre-therapy FLR function was 2.0%/min/m^2^ (IQR 1.7–2.3) in PVE-patients and 1.6%/min/m^2^ (IQR 1.1–2.4) in RL-patients (*p* = 0.401). Definitions of the FLR and additional pre-therapy functions and volumes are provided (Table 1).

**Table 1:** Baseline characteristics

		PVE	*n*	RL		*n*	*p* value	
		Median (IQR)	10			10		
Age at treatment		65.5 (58.8–72.5)		59.5 (50.3–68.8)			0.256	
Largest liver lesion (mm)		23.0 (9.6–47.4)		45.6 (30.8–67.7)			0.059	
Tumour distribution		Unilobar 40% (4)		Unilobar 40% (4)				
		Bilobar 60% (6)		Bilobar 60% (6)				
Pre-therapy FLR function		2.0 (1.7–2.3)		1.6 (1.1–2.4)			0.401	
Pre-therapy FLR volume		504.5 (289.5–567.0)		456.5 (315.8–517.0)			0.650	
Pre-therapy total liver function		7.2 (5.9–8.2)		6.6 (5.4–9.8)			0.880	
Pre-therapy total liver volume		1486.0 (1357.8–1865.0)		1557.0 (1262.5–2287.0)			0.705	
CCI		8 (7.5–9.0)		8 (6.8–8.5)			0.504	
		% (n)		% (n)				
Male		60% (6)		80% (8)			0.628	
Chemo prior to treatment		90% (9)		90% (9)			1.000	
FLR								
S1-3		20% (2)		30% (3)				
S1-4		60% (6)		40% (4)				
S2-3		10% (1)		–				
S2-4		10% (1)		10% (1)				
S5-7		–		10% (1)				
S6-7		–		10% (1)				

*CCI* Charlson comorbidity index, *FLR* future liver remnant, *IQR* interquartile range, *n* number, *PVE* portal vein embolization, *RL* radiation lobectomy, *S* segment

### Response to treatment

**Fig. 3:**
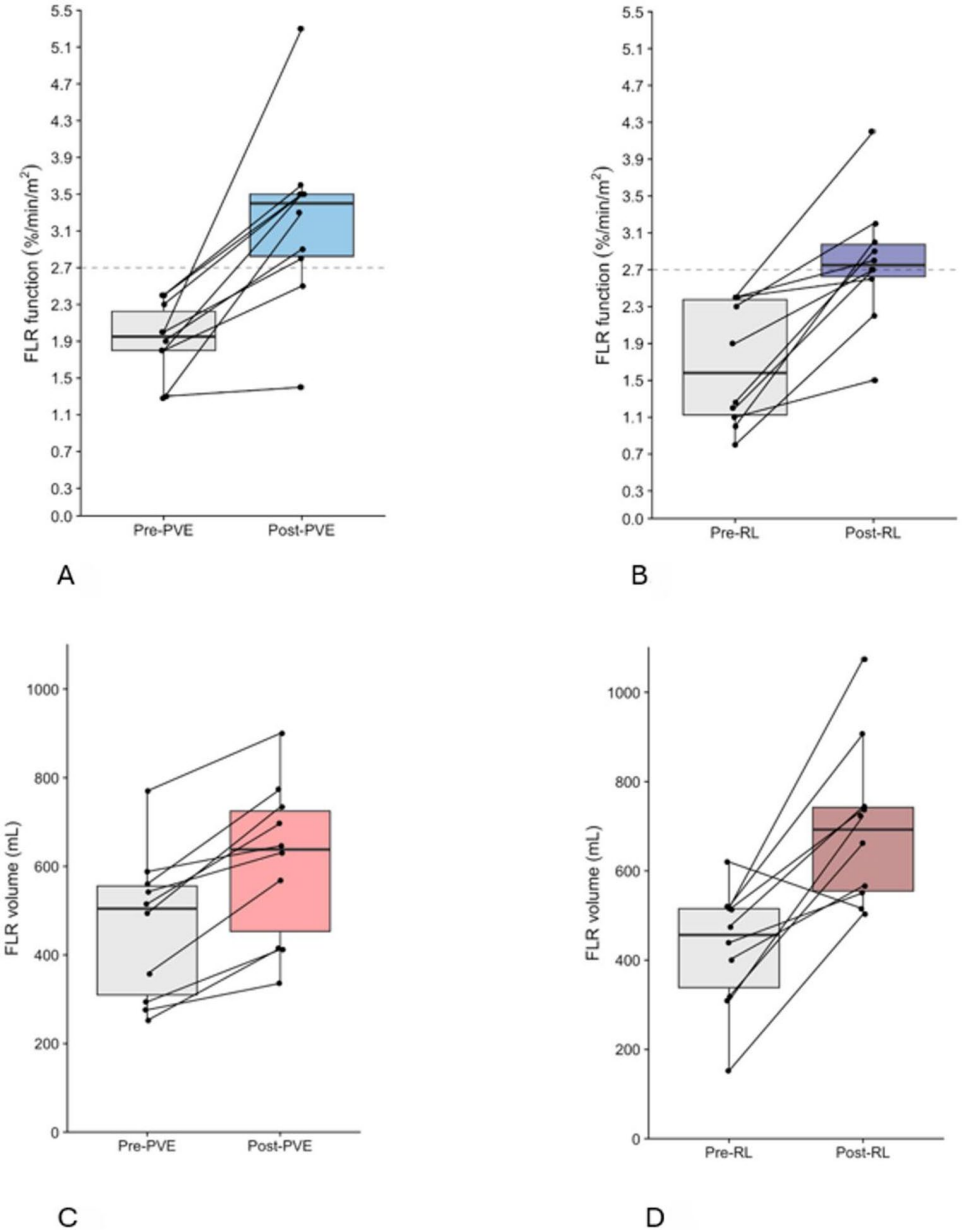
FLR response data. **A** Functional increase after PVE, **B** functional increase after RL, **C** voluminal increase after PVE, **D** voluminal increase after RL

Both groups of patients treated with PVE and RL had a median absolute increase of the FLR function of 1.2%/min/m^2^ (*p* = 0.001 and *p* < 0.001, respectively). However, patients treated with RL had a – albeit non-significant - lower pre-treatment function compared to PVE (Tables 1 and 2; Fig. 3). In total, four out of ten patients in the RL cohort started with a pre-treatment FLR function below 1.72%/min/m^2^ and nevertheless reached the functional threshold of 2.7%/min/m^2^ (Table 1, Supplementary Table 2).

**Table 2:** Treatment response. Statistical significance was defined as *p* < 0.05

	Pre-treatment		Post-treatment		Increase		
	Median (IQR)	*n*	Median (IQR)	*n*	Median (IQR)	*n*	*p* value
PVE		10		10		10	
FLR function	2.0 (1.7–2.3)		3.4 (2.7–3.5)		1.2 (0.8–1.8)		**0.001**
Total liver function	7.2 (5.9–8.2)		6.8 (6.1–8.0)		− 0.25 (− 1.6–1.0)		0.751
FLR volume	504.5 (289.5–567.0)		638.0 (414.3–744.0)		146.5 (146.5–211.8)		**< 0.001**
Total liver volume	1486.0 (1357.8–1865.0)		1506.5 (1395.8–1790.0)		42.5 (− 106.3–180.3)		0.686
RL							
FLR function	1.6 (1.1–2.4)		2.8 (2.5–3.1)		1.2 (0.4–1.7)		**< 0.001**
Total liver function	6.6 (5.4–9.8)		5.6 (5.0–9.8)		− 1.4 (− 2.0- -0.3)		**0.047**
FLR volume	456.5 (315.8–517.0)		692.5 (542.0–784.8)		307.0 (152.5–393.8)		**0.001**
Total liver volume	1557.0 (1262.5–2287.0)		1748.0 (1471.5–2361.3)		93.5 (− 64.8–268.0)		0.095

*FLR* future liver remnant, *IQR* interquartile range, *n* number, *PVE* portal vein embolization, *RL* radiation lobectomy

**Fig. 4:**
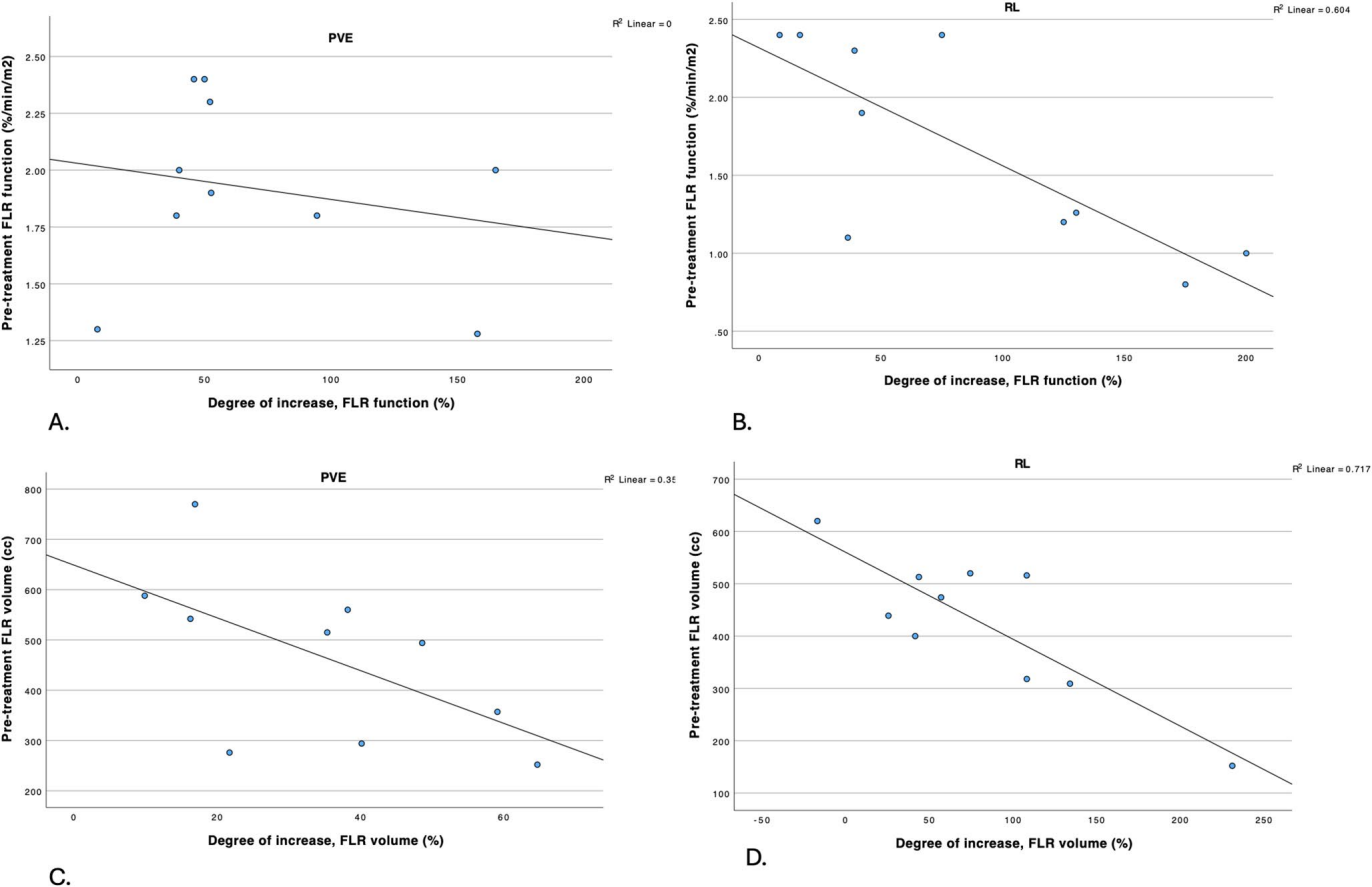
Correlation between pre-therapy FLR function and degree of functional increase in groups. **A** PVE-cohort, **B** RL- cohort. Correlation between pre-therapy FLR volume and degree of voluminal increase in groups. **C** PVE-cohort, **D** RL- cohort

PVE-treated patients showed a median degree of functional increase of 51.1% (IQR 39.7–110.3), compared to 58.6% (IQR 31.4–141.4) among those who received RL (*p* = 0.940). A sufficient function of the FLR was achieved faster after PVE than after RL (median 31 days (IQR: 28–54.5) and 75 days (IQR 64–85) respectively, *p* = 0.002). FLR growth rates per day were calculated to correct for these differences in duration of follow-up. A significantly higher median functional growth rate per day was observed after PVE compared to RL (0.029 (IQR 0.017–0.039) versus 0.014 (IQR 0.006–0.021), respectively (*p* = 0.028)) (Table 3). In addition, for RL-patients, the pre-therapy FLR function correlated significantly with the degree of increase, i.e. the smaller the pre-treatment FLR function, the bigger the degree of increase (pearson’s correlation coefficient: − 0.777, *p* = 0.008) (Figure 4; Table 4).

**Table 3:** Percentual increase and growth rate. Statistical significance was defined as *p* < 0.05

	PVE		RL		
	Median (IQR)	*n*	Median (IQR)	*n*	*p* value
% hypertrophy		10		10	
FLR function	51.1% (39.7–110.3)		58.6% (31.4–141.4)		0.940
Total liver function	− 3.5% (− 20.2–16.7)		− 22.1% (− 27.9–5.9)		0.082
FLR volume	36.8% (16.7–51.2)		65.7% (37.5–114.6)		**0.049**
Total liver volume	− 0.5% (− 6.6–16.7)		5.9% (− 3.5–17.2)		0.450
Growth rate/day					
FLR function	0.029 (0.017–0.039)		0.014 (0.006–0.021)		**0.028**
Total liver function	0.004 (− 0.026–0.033)		− 0.011(− 0.022–0.014)		0.496
FLR volume	3.538 (2.019–6.080)		3.875 (1.828–5.254)		0.821
Total liver volume	− 0.100 (− 3.472–3.253)		1.304 (− 0.5203.229)		0.364
Follow up time					
Treatment-imaging (days)	31 (28–54.5)	10	75 (64–85)	10	**0.002**

*FLR* future liver remnant, *IQR* interquartile range, *n* number, *PVE* portal vein embolization, *RL* radiation lobectomy

**Table 4:** Correlation analyses per group. Statistical significance was defined as *p* < 0.05

	PVE	RL
Pre-therapy FLR-function and degree of increase (function)		
Correlation coefficient	− 0.208	− 0.777
* p* value	0.565	**0.008**
Pre-therapy FLR-volume and degree of increase (volume)		
Correlation coefficient	− 0.847	− 0.593
* p* value	**0.002**	0.071

The median absolute increase of volume was significant in both groups (PVE: 146.5 cc (IQR: 146.5–211.8) *p* < 0.001), RL: 307.0 cc (IQR: 152.5–393.8) *p* = 0.047). The degree of voluminal increase was significantly higher in the RL group versus the PVE group; (65.7% (IQR 37.5–114.6) versus 36.8% (IQR 16.7–51.2) *p* = 0.049), respectively (Tables 2 and 3).

**Fig. 5:**
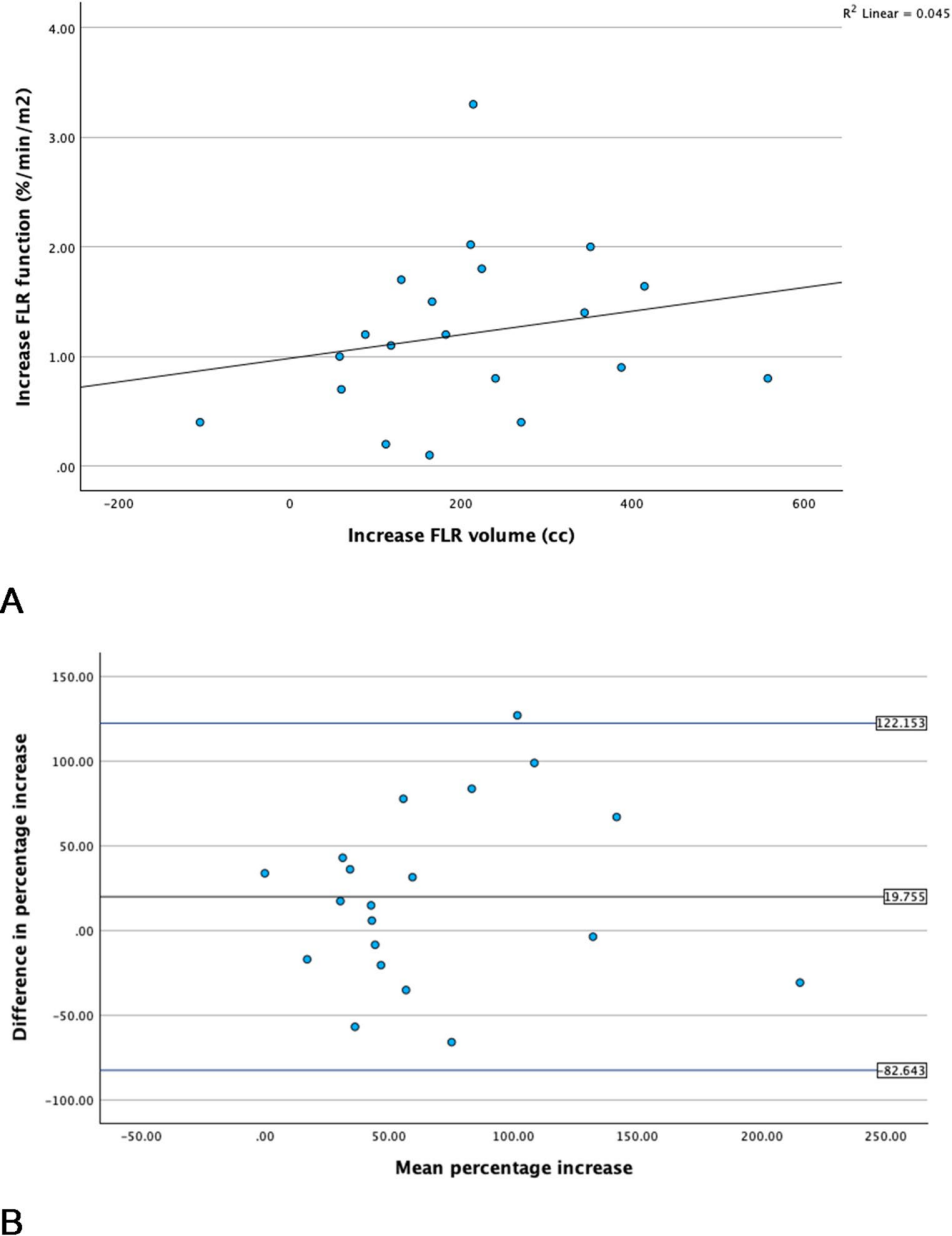
Absolute FLR functional and voluminal changes between baseline and follow-up **A** Scatter plot, **B** Bland-Altman plot

A scatter plot of all patients illustrates the relation between the percentual increase in FLR function and volume. A positive but weak correlation was observed (*r* = 0.213, *p* = 0.367). In addition, a Bland-Altmann was performed to assess the agreement between functional and voluminal increase. The mean difference in percentual increase was 19.8 and limits of agreement ranged from − 82.6 to 122.2 (Figure 5).

### Perioperative characteristics

Nine (90%) of PVE-treated patients underwent surgical treatment. One patient could not proceed to surgery, due to insufficient growth of the FLR (1.3%/min/m^2^ pre-treatment and 1.4%/min/m^2^ 28 days after treatment). In the RL-cohort, major hepatic resection could not be performed in four patients (40%). The first patient did undergo surgery but was deemed irresectable during the procedure due to extrahepatic disease progression. The remaining three patients were ineligible for surgery because of disease progression both inside the FLR and outside the liver. Two of these patients failed to achieve sufficient FLR function as well. Types of surgery are further specified in Table 5.

**Table 5:** Perioperative characteristics. Statistical significance was defined as *p* < 0.05

	PVE (*n* = 10)	RL (*n* = 10)	
	% (*n*)	% (*n*)	*p* value
**Proceed to surgery**	**90% (9)**	**70% (7)**	0.303
Right lobectomy	33.3% (3)	33.3% (2)	
Extended right lobectomy	22.2% (2)	33.3% (2)	
Right lobectomy + wedge segment 2	11.1% (1)	–	
Right lobectomy + wedge segment 3	11.1% (1)	–	
Right lobectomy + wedge segment 4	22.2% (2)	–	
Extended left lobectomy	–	16.7% (1)	
Left lobectomy + segment 8	–	16.7% (1)	
Resection	90% (9)	60% (6)	
R0	77.8% (7)	83.3% (5)	1.000
R1	22.2% (2)	16.7% (1)	
R2	0% (0)	0% (0)	
Mortality			
In hospital	0% (0)	0% (0)	
30 days	0% (0)	0% (0)	
90 days	0% (0)	0% (0)	
PHLF			
0	75% (6)	33.3% (2)	
A	12.5% (1)	66.7% (4)	
B	12.5% (1)	0% (0)	
C	0% (0)	0% (0)	
	Median (IQR)	Median (IQR)	*p* value
Length of stay	7.0 (5.5–10.5)	10.5 (7.0–17.0)	0.122
Days: treatment-surgery	73.0 (49.5–79.0)	104.0 (89.0–123.0)	**0.007**

*d* day, *min* minimum, *max* maximum, *n* number, *PVE* portal vein embolization, *RL* radiation lobectomy, *SD* standard deviation

The length of in-hospital stay after surgery did not differ significantly between groups. The median time between treatment and surgery was longer for RL-treated patients (104 days (IQR: 89–123) versus 73 days (IQR 49.5–79) after PVE, *p* = 0.007). In both groups, R0 resection margins could be accomplished in most cases (7/9 in PVE-, 5/6 in RL-treated patients) and R2 resection margins did not occur. No in hospital, 30-day or 90-day mortality was observed in the entire cohort.

PHLF occurred in two PVE-treated patients (25%) and four RL-treated patients (66.7%). One PVE-treated patient developed severe ascites for which a diagnostic abdominal aspiration and CT-abdomen were performed (grade B). The remaining PVE-patient with PHLF was classified as grade A, as the patient did not require additional treatment nor deviations from the regular post-hepatectomy treatment protocol. Similarly, in the RL-group, all cases of PHLF were classified as grade A (Table 5).

## Discussion

PVE and RL induce hypertrophy of the FLR through fundamentally different mechanisms. PVE is based on vascular occlusion of the portal branches supplying the liver segments planned for resection. By redirecting portal flow toward the FLR, PVE triggers a cascade of hepatocellular proliferation, sinusoidal remodeling, and inflammatory signaling that together stimulate compensatory hypertrophy (Figure 1A) [26]. This mechanism is well studied and has long formed the basis of PVE as the standard preoperative strategy to enhance FLR capacity [27]. In contrast, RL induces hypertrophy through the arterial route by targeting the predominantly arterially fed tumor-bearing lobe. By delivering selective internal radiation, RL achieves local tumor control while simultaneously creating progressive functional decline in the treated lobe. The contralateral lobe responds with hypertrophy, but, interestingly, the exact biological pathway remains ununderstood (Figure 1B). Current hypotheses include radiation-induced parenchymal atrophy, altered intrahepatic hemodynamics, microvascular injury, and cytokine-mediated regenerative signaling [28]. Although the mechanistic basis of RL driven hypertrophy is not understood, increasing evidence suggests that its regenerative effect is both clinically meaningful and sustained over a longer timeframe compared to PVE [29, 30]. Moreover, irradiation additionally allows RL to function as a clinical-oncological “test-of-time”, identifying patients with biologically aggressive disease during the hypertrophy period and thereby potentially preventing futile surgery [28].

The mechanistic differences between PVE and RL translate directly into their clinical performance. PVE induces rapid hypertrophy through abrupt redirection of portal flow towards the FLR [27], which explains why PVE typically results in predictable hypertrophy within 3–6 weeks and consistently increases resectability rates in real-world patients cohorts. A systematic review revealed a mean voluminal increase of the FLR of 37.9% (± 0.1%) after a mean response interval of 25.9 days [12]. On the other hand, RL produces slower volumetric gains, driven by radiation induced atrophy of the treated contralateral lobe, while simultaneously providing local tumor control. Reported volumetric outcomes range from 26% to 45% increase after a period of 44 days to 9 months [16–19]. These differences yield complementary strengths, PVE remains the preferred strategy when a rapid increase in FLR is required, and oncologic risk is limited. RL, however, may be advantageous in patients who benefit from concurrent tumor control, those with borderline resectability, or when PVE is technically infeasible [30, 31].

This retrospective cohort study evaluated FLR response after portal vein embolization PVE and RL in patients with CRLM. In this study, RL achieved functional hypertrophy comparable to PVE; the median functional increase was 51.1% (IQR 39.7–110.3) after PVE and 58.6% (IQR 31.4–141.4) after RL (*p* = 0.940), supporting prior literature demonstrating the regenerative capacity of both interventions [11]. Although hypertrophy developed more slowly after RL (median 75 days after RL vs. 31 days after PVE, *p* = 0.002), ultimate functional outcomes were similar, supporting RL as a valid alternative when PVE is technically challenging or when oncologic considerations favor RL, such as borderline resectability, vascular involvement, or need for tumor control during the waiting interval. i.e. “test-of-time”. In this study, 40% of RL patients were deemed unresectable due to progression during follow-up. It should be noted, however, that patients in the TARE group had more advanced disease at baseline, which could have influenced both resectability and oncologic outcomes.

Volumetric results differed from Garlipp et al., who reported greater hypertrophy after PVE than RL [21]. In contrast, the present study observed higher volumetric gains after RL (65.7% versus 36.8% after PVE, *p* = 0.049). Differences in timing of the assessment of the FLR likely explain this discrepancy, as RL-induced hypertrophy can continue for months [17] and follow-up in Garlipp’s cohort was shorter.

Moreover, functional and volumetric FLR changes showed only a weak correlation (*r* = 0.212) with minimal agreement, indicating that functional regeneration may outpace structural hypertrophy. This supports the use of hepatobiliary scintigraphy (HBS) alongside volumetry, as volume alone may underestimate true hepatic capacity. Indeed, nearly half of the included patients had a pre-treatment FLR volume > 30% yet insufficient function (< 2.7%/min/m^2^), consistent with guideline recommendations for functional imaging [32]. This was further confirmed by findings from Allimant et al., who studied dynamic liver function using HBS after glass 90Y radioembolization. Their findings revealed early functional decline in the treated lobe, followed by recovery after approximately six months, emphasizing that functional changes do not always parallel volumetric hypertrophy and that volumetry alone may misrepresent true hepatic regeneration [33]. Lastly, an inverse relationship between baseline FLR volume and hypertrophy was observed, aligning with prior work [34]. Notably, patients treated with RL began with lower FLR function, yet most patients with pre-treatment function < 1.72%/min/m^2^ still achieved resection thresholds. As a major clinical implication, this suggests that RL may benefit patients with critically low starting function, whereas Cieslak et al. [15] demonstrated that specific PVE patients require a pre-treatment function of at least 1.72%/min/m^2^ to achieve sufficient FLR function.

Limitations include the retrospective design, small sample size, and different follow-up intervals between PVE and RL, which may have delayed recognition of sufficient hypertrophy in RL patients. Variability in embolization materials and RL dosing further limits standardization. Despite these limitations, this study provides evidence that RL yields functional outcomes comparable to PVE while offering distinct oncologic advantages. The findings underscore the importance of functional assessment and support RL as a valuable tool for FLR augmentation, warranting further prospective investigation.

Future work should focus on optimizing RL, particularly through ongoing studies that focus on dose-escalation strategies and individualized activity planning [35, 36]. As RL dosing remains heterogenous across centers, it is essential to determine the optimal balance between hypertrophy induction, safety and tumor control. In parallel, the biological mechanisms underlying RL induced hypertrophy remain poorly understood. Current hypotheses are largely extrapolated from retrospective clinical series, underscoring the need for research using clinical specimens, advanced animal models, organoids and organ on chip models. Comparative prospective studies are also needed to clarify the relative clinical performance of PVE versus RL and to further investigate the hypothesis that RL might be able to induce sufficient functional gain in patients starting from lower baseline FLR function. Furthermore, comprehensive follow-up schedules with more timepoints could help to clarify functional growth patterns after RL.

## Conclusion

This study demonstrates that RL can achieve FLR hypertrophy comparable to PVE in patients with CRLM prior to surgical treatment, albeit with a slower regenerative trajectory. Functional assessment revealed clinically relevant liver regeneration that was not reliably reflected by volumetric changes, highlighting the importance of incorporating hepatobiliary scintigraphy into preoperative evaluation. In addition, both pre-operative treatment options were safe; no major post-surgical morbidities or 90-day mortality were observed in the entire cohort. Together, these findings support RL as a valuable alternative to PVE, particularly in patients with low baseline liver function, borderline resectability, or a need for simultaneous tumor control. Future prospective studies are warranted to optimize treatment protocols and clarify the biological basis of radiation-induced liver regeneration.

## Electronic Supplementary Material

Below is the link to the electronic supplementary material.

Supplementary Material 1

## Data Availability

The data that support the findings of this study are available upon reasonably request via contacting the corresponding author.
