# Supplementary material for: Portal vein embolization versus radiation lobectomy as pre-treatment for major liver resection for colorectal liver metastases: functional assessment of the future liver remnant
Source: Angiogenesis. 2026 May 20;29(3):34. doi: 10.1007/s10456-026-10049-5 (PMC13190528; doi:10.1007/s10456-026-10049-5)
Supplement: Supplementary file 1 — Supplementary Material 1 [file 10456_2026_10049_MOESM1_ESM.docx]

**Supplementary appendix**

| **Portal vein embolization** | Method | Portal segments embolized | **Total volume FLR (cc)** | | **Total FLR volume % increase** | **Function FLR** | | **Total FLR Function % increase** |
| --- | --- | --- | --- | --- | --- | --- | --- | --- |
| Patiënts |  |  | Pre-PVE | Post- PVE |  | Pre-PVE | Post- PVE |  |
| **1** | PVA particles & coils | 4-8 | 357.0 | 568.0 | 59.1 | 1.3 | 3.3 | 157.8 |
| **2** | Contour particles & coils | 5-8 | 252.0 | 415.0 | 64.7 | 1.3 | 1,4 | 7.7 |
| **3** | Contour particles & amplatzer plug | 5-8 | 588.0 | 646.0 | 9.9 | 1.9 | 2.9 | 52.6 |
| **4** | PVA particles & coils & amplatzerplug in right hepatic vein | 5-8 | 276.0 | 336.0 | 21.7 | 1.8 | 2.5 | 38.9 |
| **5** | PVA particles & coils & amplatzerplug | 5-8 | 515.0 | 697.0 | 35.3 | 2.4 | 3.6 | 50.0 |
| **6** | Contour particles & coils | 5-8 | 542.0 | 630..0 | 16.2 | 2.3 | 3.5 | 52.2 |
| **7** | PVA particles & coils & amplatzerplug | 5-8 | 294.0 | 412.0 | 40.1 | 2.4 | 3.5 | 45.8 |
| **8** | NBCA-MS (Glubran) | 5-8 | 770.0 | 900.0 | 16.9 | 1.8 | 3.5 | 94.4 |
| **9** | NBCA-MS (Glubran) | 5-8 | 560.0 | 774.0 | 38.2 | 2.0 | 5.3 | 165.0 |
| **10** | NBCA-MS (Glubran) | 5-8 | 494.0 | 734.0 | 48.6 | 2.0 | 2.8 | 40.0 |

**Table S1.** PVE characteristics and FLR response

| **Radiation Lobectomy** | Arterial segments treated | Microspheres | Activity  Administered in mBq | **Total volume FLR(cc)** | | **Total FLR volume % increase** | **Function FLR** | | **Total FLR Function % increase** |
| --- | --- | --- | --- | --- | --- | --- | --- | --- | --- |
| Patiënts |  |  |  | Pre-RL | Post- RL |  | Pre-RL | Post-RL |  |
| **1** | 4-8 | ^90^Y | 2964 | 152.0 | 503.0 | 230.9 | 1.0 | 3.0 | 200.0 |
| **2** | 4- 8 | ^90^Y | 2816 | 318.0 | 662.0 | 108.2 | 0.8 | 2.2 | 175.0 |
| **3** | 4(b)- 8 | ^166^Ho | 2841 | 400.0 | 566.0 | 41.5 | 1.2 | 2.7 | 125.0 |
| **4** | 1-4, 8 | ^90^Y | 1056 | 620.0 | 515.0 | -16.9 | 2.4 | 2.8 | 16.7 |
| **5** | 5-8 | ^166^Ho | 2996 | 439.0 | 551.0 | 25.5 | 2.4 | 2.6 | 8.3 |
| **6** | 2-5, 8 | ^90^Y | 2086 | 516.0 | 1074.0 | 108.1 | 1.9 | 2.7 | 42.1 |
| **7** | 5-8 | ^90^Y | 4337 | 520.0 | 907.0 | 74.4 | 2.3 | 3.2 | 39.1 |
| **8** | 4-8 | ^90^Y | 4103 | 474.0 | 744.0 | 56.9 | 1.1 | 1.5 | 36.4 |
| **9** | 5-8 | ^166^Ho | 5470 | 309.0 | 723.0 | 133.9 | 1.3 | 2.9 | 130.2 |
| **10** | 5-8 | ^166^Ho | 5000 | 513.0 | 737.0 | 43.7 | 2.4 | 4.2 | 75.0 |

**Table S2.** RL characteristics and FLR response
